# Next Generation Sequencing for Potential Regulated Genes and Micro-RNAs of Early Growth Response-1 in the Esophageal Squamous Cell Carcinoma

**DOI:** 10.1007/s10930-022-10079-0

**Published:** 2022-10-07

**Authors:** Yen-Chiang Tseng, Chih-Wen Shu, Hui-Min Chang, Yi-Hsuan Lin, Yen-Han Tseng, Han-Shui Hsu, Yih-Gang Goan, Ching-Jiunn Tseng

**Affiliations:** 1 Division of Thoracic Surgery, Department of Surgery, Kaohsiung Veterans General Hospital, Kaohsiung, Taiwan; 2 Institute of Clinical Medicine, National Yang Ming Chiao Tung University, Taipei, Taiwan; 3 Division of Thoracic Surgery, Department of Surgery, Taipei Veterans General Hospital, Taipei, Taiwan; 4 Institute of BioPharmaceutical Sciences, National Sun Yat-Sen University, No. 70, Lianhai Rd., Gushan Dist, Kaohsiung, 80424 Taiwan; 5 Department of Biomedical Science and Environmental Biology, Kaohsiung Medical University, Kaohsiung, 80708 Taiwan; 6 Department of Medical Education and Research, Kaohsiung Veterans General Hospital, No. 386, Dazhong 1st Rd., Zuoying Dist, Kaohsiung, 81362 Taiwan; 7 Department of Family Medicine, School of Medicine, National Yang Ming Chiao Tung University, Taipei, Taiwan; 8 Department of Public Health, College of Public Health, National Taiwan University, Taipei, Taiwan; 9 Department of Chest Medicine, Taipei Veterans General Hospital, Taipei, Taiwan; 10 School of Medicine, National Yang Ming Chiao Tung University, Taipei, Taiwan; 11 Institute of Emergency and Critical Care Medicine, National Yang Ming Chiao Tung University, Taipei, Taiwan

**Keywords:** EGR1, Tumor suppressor, Esophageal carcinoma

## Abstract

Esophageal cancer has a poor prognosis due to its aggressiveness and low survival rate. In Ease Asia, esophageal squamous cell carcinoma (ESCC) outnumbers esophageal adenocarcinoma (EAC). The ESCC patients still have high mortality despite modern surgical resection and neoadjuvant treatment. Determining patient and outcome prognostic factors is critical in ESCC treatment. In esophageal cancer, early growth response-1 (Egr-1) is a tumor suppressor gene, but the mechanism and associated genes are unknown. The study utilizes RNA interference method, the platform of Next Generation Sequencing (NGS) and bioinformatics analysis to investigate the influences after the Egr-1 gene slicing on the ESCC cells. The heat maps of differentially expressed mRNA and microRNAs were analyzed using the algorithm, Burrows-Wheller Aligner. The study showed that the expression of 51 mRNA and 26 microRNAs have significant changes in ESCC cells after Egr-1 knockdown. The KEGG enrichment analysis linked Egr-1-regulated genes and microRNAs. Egr-1 interactions with these genes and microRNAs may be important in tumor progression. In conclusions, this study provided the transcriptome patterns and relating pathway analysis for Egr-1 knockdown in ESCC cells. The mRNA and microRNAs altered by Egr-1 gene silencing might provide key information in the treatment of ESCC.

## Introduction

Esophageal cancer is associated with poor survival rate despite surgical resection. Two main histological subtypes, esophageal squamous cell carcinoma (ESCC) and esophageal adenocarcinoma (EAC), have significant differences in epidemiology, etiology, and treatment response. In East Asia and Africa, ESCC is the predominant subtype. Many clinicopathological variables, included the depth of tumor invasion, lymph node involvement, lymphovascular invasion, intramural metastasis, and the stage of disease, have been examined to predict the prognosis. Multiple molecular changes have also been investigated to elucidate the mechanism of ESCC tumorigenesis [1–3]. In recent years, neoadjuvant treatment, including neoadjuvant chemotherapy (nCT) or neoadjuvant chemoradiotherapy (nCRT), followed by esophagectomy, has become the standard strategy for resectable locally advanced esophageal cancer due to the survival benefit. Moreover, the pathological response after nCT or nCRT has been demonstrated to be independently associated with overall survival [4, 5]. Therefore, it is important to set up a new system to predict the pathological response and survival rate.

The early growth response-1 (Egr-1) is a zinc-finger transcription factor of 59 Kilodaltons. Egr-1 is involved in the regulation of cell growth and differentiation in response to signals, such as mitogens, growth factors, and stress stimuli [6]. Analysis of certain human tumor cells and tissues indicate that Egr-1 acts as a tumor suppressor [7–9]. Egr-1 suppress the function of 4E-BP1, which in turn sequester eIF4E and lead to rapamycin insensitivity.

Gao et al. showed that miRNA-191 modulates Egr-1 and the prognosis of ESCC [10]. Zhao et al. showed that Egr-1 represses ESCC through ERK1/2 signaling pathway [11]. Other studies revealed miRNAs are involved in the origin and development stage of ESCC as well as chemosensitivity [12–15]. These articles indicate that Egr-1 play important roles in ESCC by controlling cell growth, proliferation, differentiation, and angiogenesis both epigenetically and genetically. However, variable miRNAs, together with different miRNAs-mRNAs regulatory systems, lead to complex outcomes that require further investigation [16].

Our preliminary data revealed that Egr-1 is an important tumor suppressor which highly corelates with chemosensitivity in ESCC (in pressing). The Egr-1 could play an important role as a tumor suppressor as well as influencing the chemotherapeutic effects. Therefore, we investigated the miRNAs linking in the Egr-1 as well as explore the relationship between Egr-1 and these miRNAs to explore the miRNAs-mRNAs regulation in ESCC.

## Materials and Methods

### Cell Culture

Esophageal cancer cell line, CE81T, were obtained from Dr. Cheng-Po Hu at Taipei Veterans General Hospital and cultured in Dulbecco’s modified Eagle’s medium (DMEM) (Invitrogen-Gibco, Carlsbad, USA), with 10% heat-inactivated fetal bovine serum (Biological Industries, Kibbutz Beit-Haemek, Israel), 100 U/mL penicillin (Invitrogen-Gibco, Carlsbad, USA), 1% MEM non-essential amino acids (NEAA) and 100 µg/mL streptomycin (Invitrogen-Gibco, Carlsbad, USA), at 37 ℃ in a humidified 5% CO_2_ atmosphere. Cells were grown in Corning tissue culture-treated plastic (Corning, Inc., Corning, USA).

### siRNA Knockdown

siRNA-mediated EGR-1 knockdown was carried out in the CE81T cell line to observe the cell growth and viability of ESCC tumor cells. The EGR-1 siRNA oligos pool (1:5′-GAUGAACGCAAGAGGCAUA-3′; 2:5′- CGACAGCAGUCCCAUUUAC-3′; 3:5′-GGACAUGACAGCAACCUUU-3′; 4:5′- GACCUGAAGGCCCUCAAUA-3′) were synthesized by Genomics BioScience and Technology Co., Ltd. (Taipei, Taiwan). All transient transfections of the siEGR-1 oligos pooled at a final concentration of 10 nM were accomplished with Lipofectamine RNAiMAX (Invitrogen, Carlsbad, CA, USA) following the manufacturer’s protocols. Esophageal cancer cells were seeded into six-well flat-bottom plates of 3 × 10^5^ cells per well-containing 1 mL of the medium. The siRNA oligonucleotides and RNAiMAX reagent were separately diluted by 100 µL of the opti-MEM medium and mixed, the mixture was then incubated for 15–20 min. The cells were incubated with the transfection medium overnight at 37 °C in a humidified atmosphere of 5% CO_2_. Cells were incubated for 24, 48, or 72 h before harvesting. Non-silencing control (NSC) was used at the same concentration of siRNAs.

### RNA Purification and Reverse Transcription

Cellular mRNA was extracted by TRIzol® following the protocol provided by the manufacturer. Briefly, culture media were removed and 1 mL TRIzol® is added into the culture plate. The plate is shaken gently to make TRIzol® lyse all the cells and a pipetman was used to pipet the cell lysate up and down several times to homogenize the mixture. After incubating 5 min in room temperature, the whole content was moved to a new 1.7 mL centrifuge tube by a pipetman. Chloroform of 0.2 mL was added into the centrifuge tube and securely capped the tube. The tube was inverted several times to homogenize the solution. After incubating 3 min, the samples were centrifugated for 15 min at 12,000 × *g* at 4℃. The upper layer of centrifugated samples were transferred into a new 1.7 mL centrifuge tube with 0.5 mL isopropanol. Solutions were centrifuged again for 10 min after 10 min incubation and the supernatants were discarded. Precipitated RNA was washed by 75% ethanol and centrifuged at 7500 × *g*, 4 °C for 5 min. Finally, RNA in tube was dried at room temperature and solubilized by RNase-free water with proper volume. RNA solution was stored under − 80 °C avoiding freeze-thaw cycle. RNA content and purity was measured by Nanodrop microvolume spectrophotometer (Thermo-Fisher).

For reverse transcription, 2 µg of total RNA was converted to cDNA using reverse transcription kit (Applied Biosystems, Foster City, CA, USA). The procedure was the same as manual instructed.

### Next Generation Sequencing

The human esophageal cancer cell line CE48T were sequenced to an average sequencing coverage of at least 300× on Illumina Hiseq 4000 platform (Illumina, San Diego, CA). The sequencing data were first demultiplexed by bck2fastq and then subjected to Trimmomatic for FASTQ file quality control (QC). Leading/trailing low quality (Phred score below 15) reads or N bases were removed. PCR duplicates were removed by Picard. Qualified reads were mapped to the reference human genome hg19 with Burrows-Wheller Aligner (BWA-mem, v0.7.12). Genome Analysis Toolkit (GATK 3.4.0) was used for local realignment around indels and base quality score recalibration. ADTEx was used to identify copy number variations (CNVs) using a normal human HapMap DNA sample NA18535. The log2 ratio cutoff value was set at ± 0.6, which excluded target genes with copy number change between 1.5 and 0.65 fold.

### Bioinformatic Analysis

The pathway related to the target genes were performed by KEGG. Target miRNA were predicted by miRbase (https://www.mirbase.org/) and Rfam (https://rfam.xfam.org/).

## Results

### Screening of Genes and MicroRNAs in the Transcriptome of the Esophageal Cancer Cell Line After Egr-1 Knockdown

**Fig. 1:**
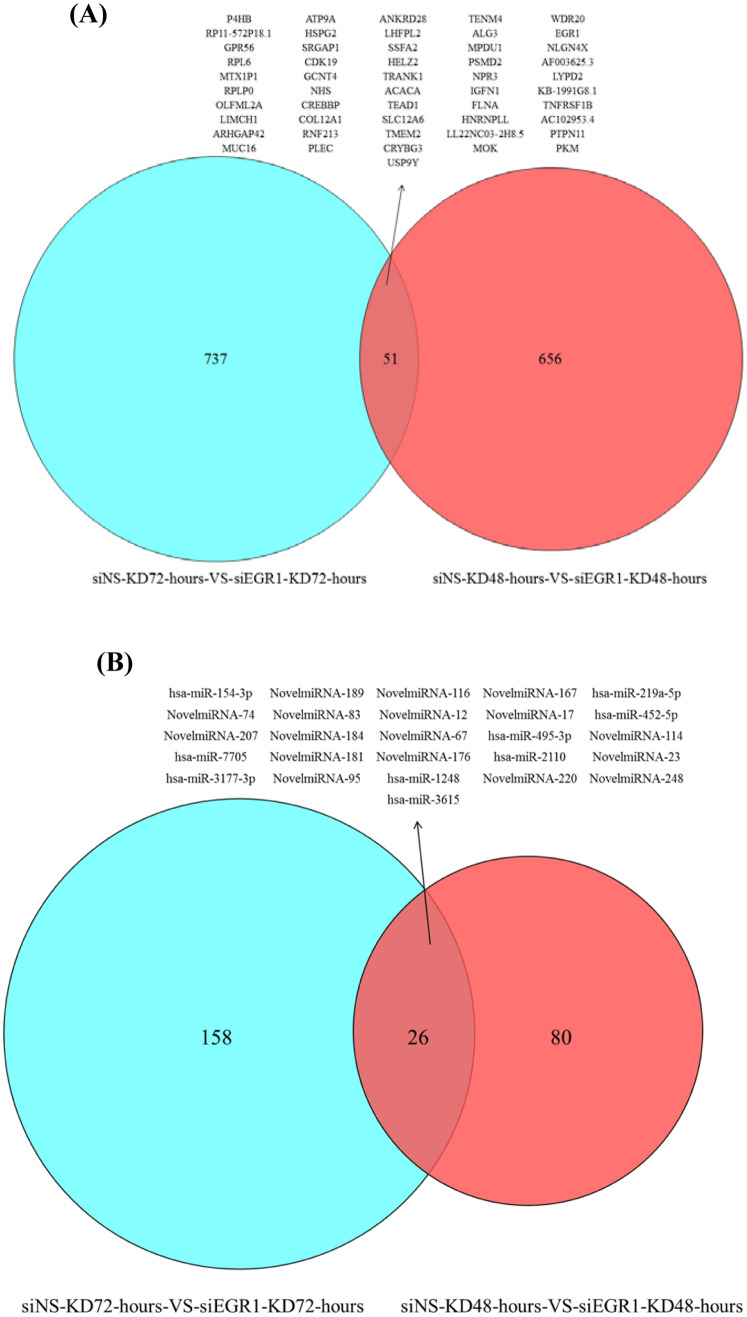
NGS was applied on the CE81T cells after Egr-1 knockdown for 48 or 72 h. The Venn diagram identified **A** 51 mRNAs and **B** 26 microRNAs with the persistent effect after gene knockdown. Knockdown is abbreviated as KD.

**Fig. 2:**
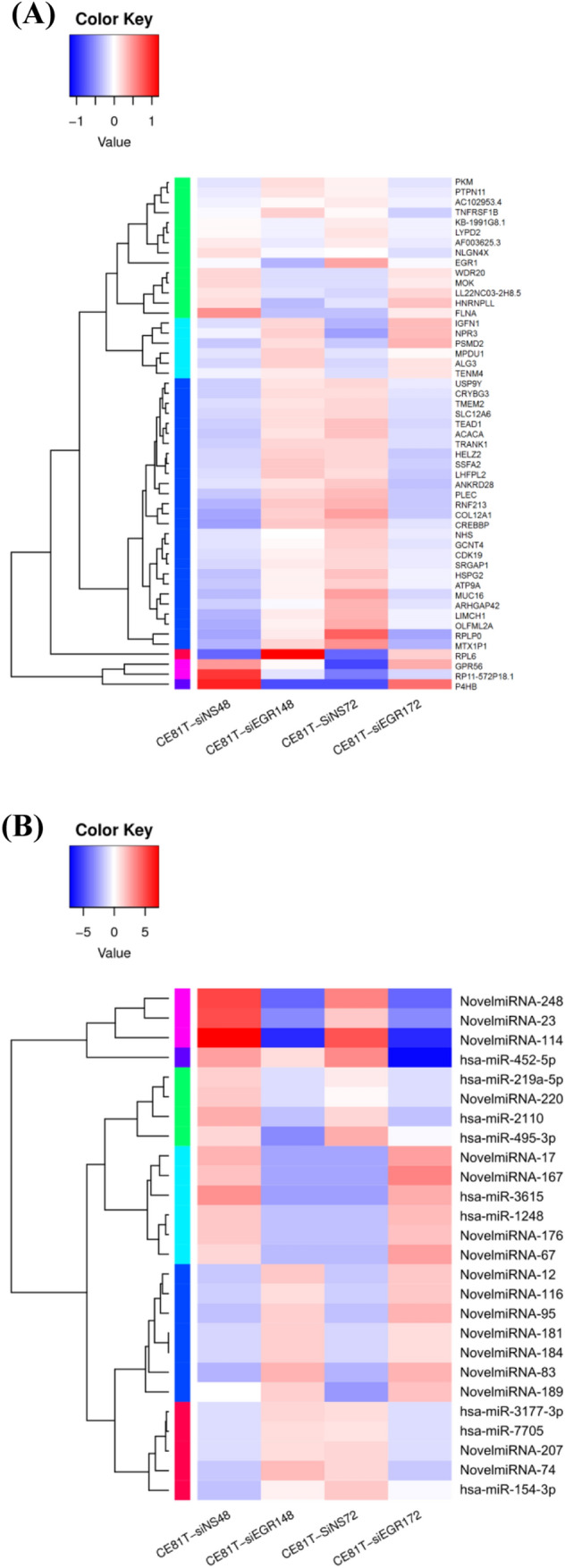
The heat maps of the **A** 51 mRNAs and **B** 26 microRNAs identified on the CE81T cells after Egr-1 knockdown for 48 and 72 h

**Fig. 3:**
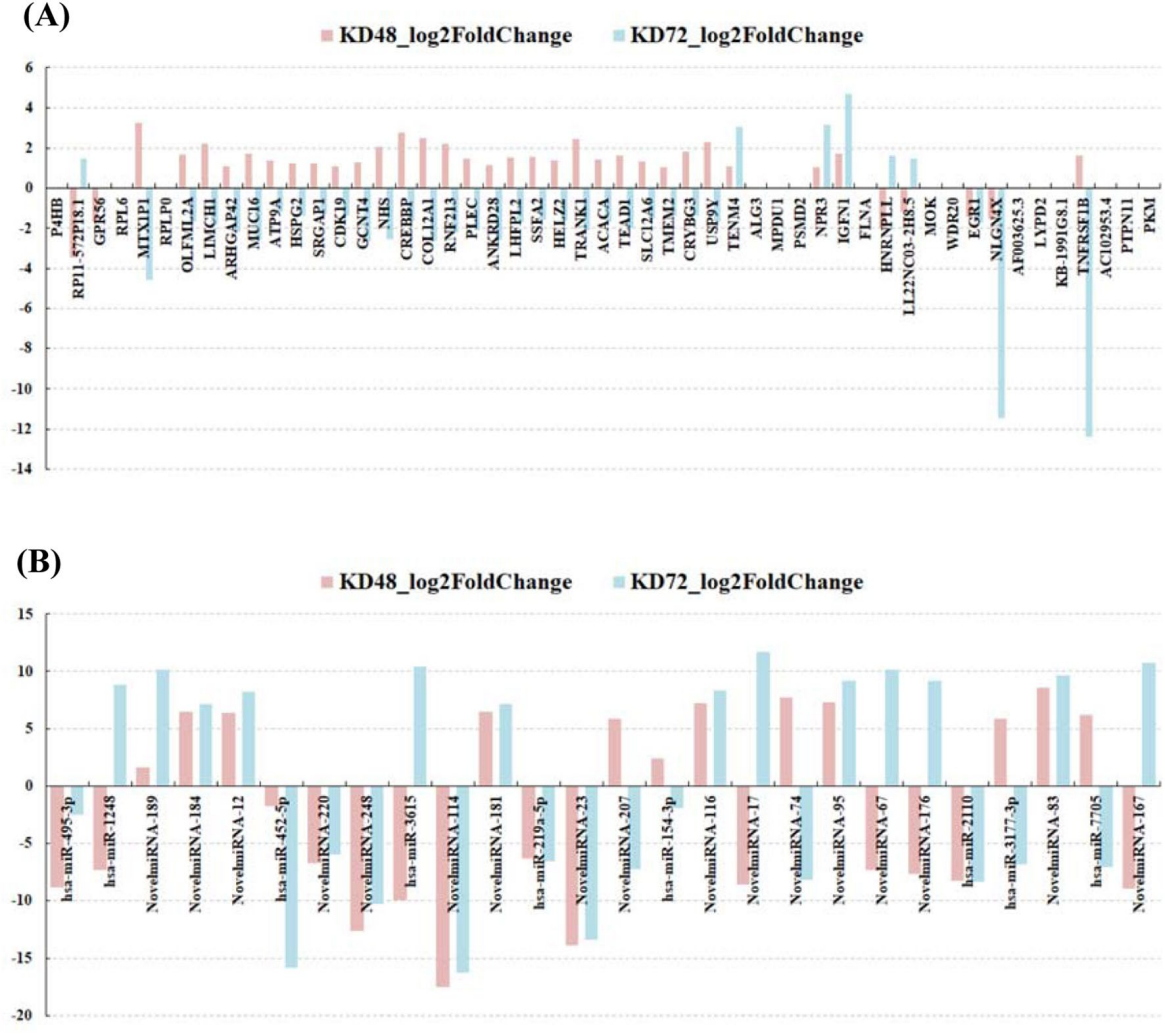
The expression levels of the **A** 51 mRNAs and **B** 26 microRNAs after Egr-1 knockdown for 48 or 72 h

To explore the mRNAs and microRNAs with overlapped expression profiles, we compared the NGS analysis between CE81T cells after Egr-1 knockdown 48 or 72 h. The 51 genes (Fig. 1A) and the 26 microRNAs (Fig. 1B) with overlapped expression patterns were determined in the NGS analysis. The cluster analysis of differentially expressed genes in the CE81T cells for the post Egr-1 knockdown was analyzed on microarray and bioinformatic analysis. Figure 2 are the heat maps of the 51 genes (Fig. 2A) and the 26 microRNAs (Fig. 2B) derived from the Egr-1 knockdown CE81T cells for 48 or 72 h. The relative fold changes of 51 genes and 26 microRNAs on the CE81T cells for the post Egr-1 knockdown 48 or 72 h was shown in Fig. 3.

### Bioinformatic Analysis for the Functions of Genes and MicroRNAs in the CE81T Cells After Egr-1 Knockdown

**Fig. 4:**
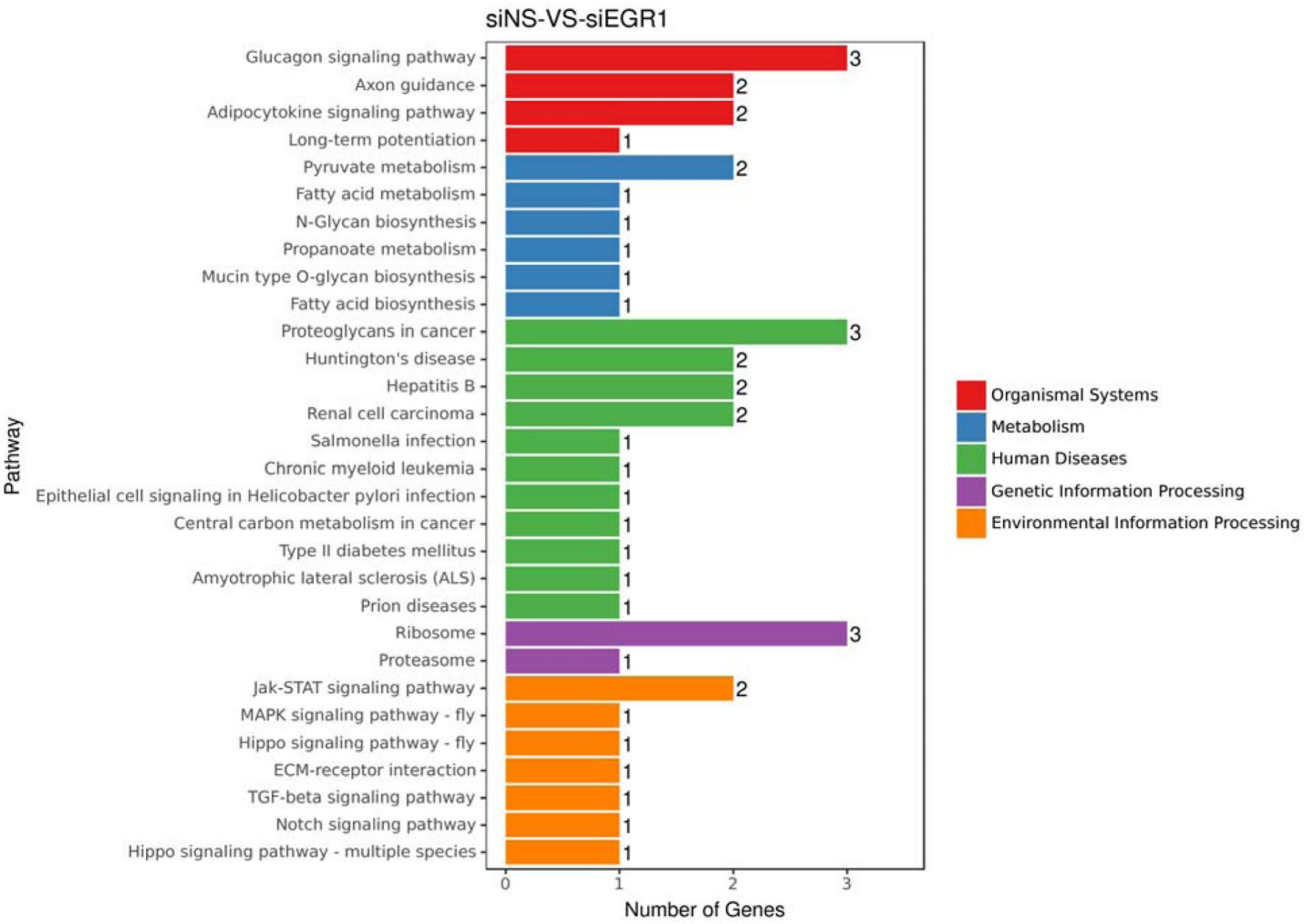
KEGG assay of the 51 identified genes after knockdown of Egr-1 with persistent effect

**Fig. 5:**
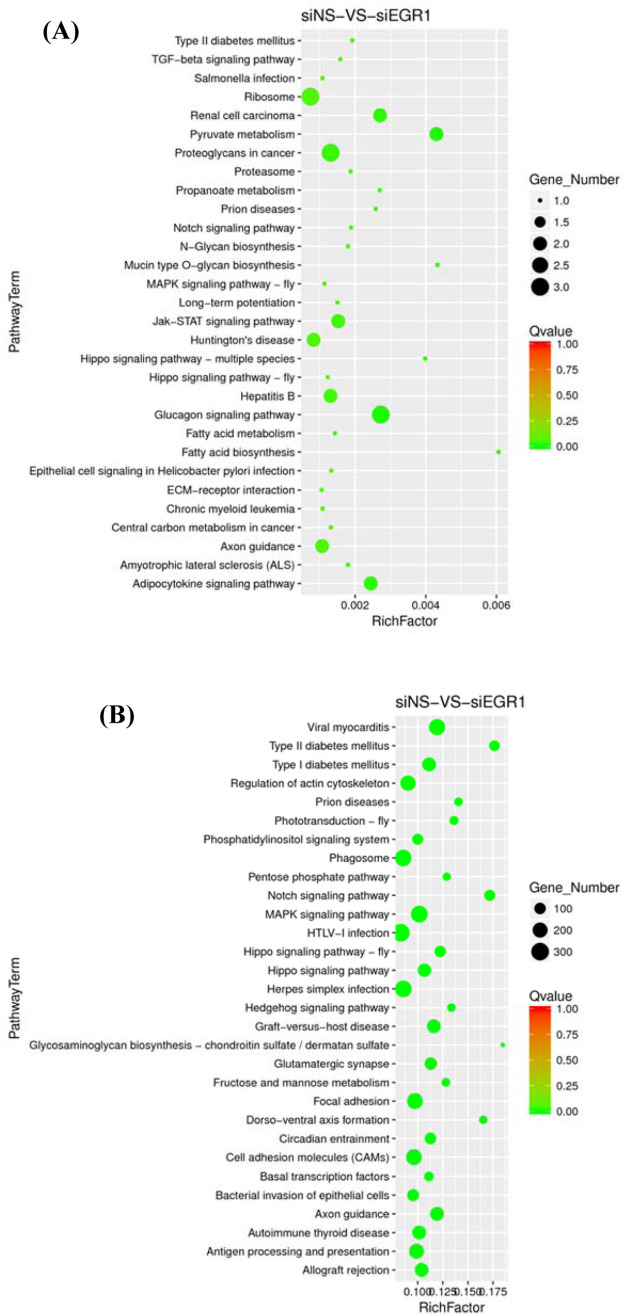
KEGG pathway enrichment bubble plot of the **A** 51 mRNAs and the **B** 26 microRNAs identified in CE81T cells after Egr-1 knockdown with persistent effect. The dot size displayed the number of genes, whereas the dot color indicated the Q-Value of the rich factors

KEGG analysis of the 51 genes in CE81T cells after Egr-1 knockdown showed that they mainly related to organismal systems, metabolism, human diseases, genetic information processing and environmental isolation processing (Fig. 4). The rich factor showed that the genes were mainly enriched in ribosome, proteoglycans in cancer, and glucagon signaling pathway (Fig. 5); while the miRNAs were mainly enriched with viral myocarditis, phagosome, MAPK signaling pathway, focal adhesion, and cell adhesion molecules (Fig. 5). We also screened out three microRNAs which interacted with the seven mRNAs with persistent effect in 48 and 72 h after knockdown of Egr-1 (Table 1).

**Table 1:** The miRNAs-mRNAs interaction identified by bioinformatics analysis

miRNA_ID	mRNA_ID	GeneID	Chr	Start	End	Strand	Gene Symbol
hsa-miR-2110	ENST00000239938	ENSG00000120738	5	1.38E + 08	1.38E + 0.8	+	EGRI
hsa-miR-3615	ENST00000278550	ENSG00000149256	11	78,363,876	79,151,992	−	TENM4
NovelmiRNA-74	ENST00000345356	ENSG00000111799	6	75,794,042	75,915,767	−	COL12A1
hsa-miR-3615	ENST00000398097	ENSG00000188158	X	17,393,543	17,754,114	+	NHS
NovelmiRNA-74	ENST00000419587	ENSG00000233280	3	97,540,884	97,594,132	+	CRYBG3
hsa-miR-3615	ENST00000467148	ENSG00000130589	20	62,189,439	62,205,592	−	HELZ2
NovelmiRNA-74	ENST00000526600	ENSG00000187079	11	12,695,969	12,966,298	+	TEAD1

Three main microRNAs interacted with seven mRNAs after Egr-1 knockdown and had persistent effect in 48 and 72 h. The hsa-miR-2110 interacted with EGR1. The hsa-miR-3615 interacted with TENM4, NHS and HELZ2. NovelmiRNA-74 interacted with TEAD1, CRYBG3 and COL12A1.

## Discussion

This is a pilot study to apply NGS to identify the interaction between mRNAs and microRNAs after EGR-1 knockdown in ESCC cells. We analyze the transcriptome which include mRNAs and small non-coding RNAs like miRNAs. Two expression pattern, Egr-1 knockdown for 48 or 72 h in ESCC cells, were compared and analyzed by bioinformatic system. The co-expression networks and predicted candidate molecules could be used to find the precision medicine for the chemotherapeutic and therapeutic targets.

Four mRNAs with the same changing pattern after Egr-1 knockdown for 48 or 72 h stand out in Fig. 3A. The expression of NLGN4X is down-regulated, but it is increased in TENM4, NPR3, and IGFN1. NLGN4X is a human specific gene for neuroligin production. Neuroligins, a family of cell adhesion molecules, are essential for synapse specification and maturation [17]. The product of TENM4 is a member of teneurin family. Teneurins can bind with latrophilin and FLRT, and direct synapse growth and formation [18, 19]. NPR3 mediates natriuretic peptides degradation, was reported to act as a tumor suppressor or promoter in some types of cancer [20, 21]. IGFN1 is a multidomain protein with more than 15 transcript variants. Aberrant splicing may lead to intronic G-quadruplex be synthesized, and lead to cancer or neurodegenerative disorder [22]. In these four genes, NLGN4X and TENM4 are adhesion molecules. Mis-regulated adhesion molecules may lead to structural deformation which may benefit cancer progression. Since there are few reports regarding the role of these four genes in esophageal cancer cell, the meaning of Egr-1 controlled gene expression still needs further studies.

Three miRNAs were sorted out with interesting interactions and worth of further research (Table 1). miRNA-2110, an onco-suppressor with the ability to suppress breast cancer and neuroblastoma [23, 24], was down-regulated after Egr-1 knockdown for 48 and 72 h. This may indicate that Egr-1 control the fate of ESCC cells by regulating the expression of miRNA-2110. After short searching, majority of the genes controlled by miRNA-3615 and NovelmiRNA-74 are for structural meaning. Those genes and two miRNAs may help to stabilize an environment which is favored by the fate of ESCC cells. Since both miRNA-3615 and miRNA-74 have different expression patterns in Egr-1 knockdown for 48 or 72 h, the real functions and effects of those two miRNAs will need more strategies to figure out.

We conducted this pilot study in CE81T cell only, which might be a problem in generalizing the results in all the ESCC cases. CE81T cell derived from the well differentiated squamous cell carcinoma of esophagus from a 57-year-old male in Taiwan. It is widely used in the research of signal transduction, apoptosis, and even pharmaceutic field, especially in Taiwan. Our results should be confirmed in other ESCC cell lines derived from human like TE-1, TE-2, and CRL-3239. The confirmed results can then serve as the foundation for the studies of ESCC treatment.

## Conclusion

There are tons of information collected after NGS sequencing. We need further evaluations and experiments to confirm the interactions between microRNAs and mRNAs. Various ESCC cell lines will be also included to determine the gene regulation by Egr-1 is consistent among different ESCC cell lines. Nevertheless, the application of NGS to identify related microRNAs as well as mRNAs after EGR-1 knockdown would be an initial point for novel prognostic factors of ESCC.
